# Do antipsychotics work in people with schizophrenia? A review of outcomes and effect sizes

**DOI:** 10.1017/S1092852925100461

**Published:** 2025-07-31

**Authors:** Christoph U Correll

**Affiliations:** 1 Department of Psychiatry, Zucker Hillside Hospital, Northwell Health, Glen Oaks, NY, USA; 2 Department of Psychiatry and Molecular Medicine, Donald and Barbara Zucker School of Medicine at Hofstra/Northwell, Hempstead, NY, USA; 3 Department of Child and Adolescent Psychiatry, Charité—Universitätsmedizin Berlin, Berlin, Germany; 4 German Center for Mental Health (DZPG), Partner Site Berlin, Berlin, Germany; 5 Einstein Center for Population Diversity (ECPD), Berlin, Germany

**Keywords:** Antipsychotics, schizophrenia, efficacy, effectiveness, tolerability, effect sizes, patient-centered care

## Abstract

**Background:**

The origins and treatment-target-related mechanisms of schizophrenia remain to be fully understood. Pharmacological and non-pharmacological treatments require expansion and improvements to meet peoples’ needs and goals. Nevertheless, antipsychotics are a cornerstone when managing schizophrenia, being essential for reducing symptom severity, preventing relapse, improving long-term functional outcomes, and reducing premature mortality risk.

**Methods:**

This narrative review synthesizes key evidence on the efficacy and risks associated with antipsychotic medications. The concept of effect sizes is introduced, allowing to compare antipsychotics across trials with different rating instruments and across different conditions.

**Results:**

The available evidence in schizophrenia and comparison with medications used for medical conditions counters the sometimes-voiced criticism that antipsychotics “do not work.” Instead, for a substantial group of people with schizophrenia, positive psychotic symptoms and global psychopathology improve witha small-medium effect size of about 0.4 versus placebo. These results are comparable to median effect sizes across commonly used medications for somatic disorders. When patients with initial response are continued on antipsychotics, the effect size increases to 0.9 for relapse prevention, translating into a number needed to treat (NNT) of about 3 to prevent one more relapse versus no treatment. This NNT is 10–20 times higher than that for the prevention of poor outcomes in some common medical conditions.

**Conclusions:**

Despite general efficacy and effectiveness of antipsychotics for schizophrenia, further development is needed regarding preventive interventions and medications with mechanisms other than postsynaptic dopamine receptor blockade, with broader efficacy for positive, negative, cognitive, suicidality, and/or reward dysregulation symptomatology, and the identification of illness mechanism/biomarker-targeting treatments to enhance treatment personalization.

## Introduction

Schizophrenia is a severe and still very often chronic and mental disorder that can profoundly affect a person’s thoughts, feelings, and behaviors.^1^ Schizophrenia impacts approximately 20 million people worldwide^2^ and often begins in late adolescence or early adulthood.^3^ Schizophrenia is characterized by a constellation of positive symptoms (hallucinations, delusions, disorganized thinking or behaviors), negative symptoms (anhedonia, avolition, amotivation, affective flattening, and alogia), cognitive dysfunction (attention, memory, speed of processing, executive functioning, social cognition), and often also mood symptoms.^1^ Without effective treatment, schizophrenia leads to substantial disability, repeated hospitalizations, social isolation, homelessness, and a 5–10% lifetime suicide risk.^1^^,^^4^

While the causes of schizophrenia are multifactorial and still only insufficiently understood, spanning genetic, neurodevelopmental, and environmental factors,^5^^,^^6^ the effective management of schizophrenia depends critically on early, sustained, and evidence-based pharmacological, psychoeducational, psychological, and psychosocial interventions.^7^^–^^10^ Among these, antipsychotics remain the cornerstone for people diagnosed with schizophrenia.^4^ It is important, though, not to confuse outcomes and treatment needs in people living with schizophrenia with those of people diagnosed with other types of psychotic disorders, including the very heterogeneous group of “first episode psychosis,” which is a mixture of disorders with varying outcomes both with and without antipsychotic treatment.^4^^,^^11^

However, antipsychotic medications are frequently the subject of public criticism and activist skepticism. Critics often cite modest trial results in moderately ill patients, high nonresponse rates, and substantial side effects to argue that antipsychotics “don’t work.”

This article provides a different view than this negative narrative with a comprehensive, accessible, and data-driven synthesis of the evidence. Drawing on relevant meta-analyses, the concept of effect sizes and real-world outcome data—including competency restoration, suicide reduction, and relapse prevention—and by comparing outcomes of antipsychotics with those of commonly used medications to treat chronic medical disorders, this article highlights that antipsychotics are among the most effective interventions in medicine for chronic illnesses.

On the other hand, clearly, gaps in antipsychotic efficacy remain.^12^ These gaps include limited efficacy of current antipsychotics for negative symptoms and cognitive dysfunction, and residual and resistant positive symptoms, despite adherence to current mechanisms of action of antipsychotics, reward dysfunction, and comorbid substance use disorders. Furthermore, although more recently approved antipsychotics have become safer and better tolerated, antipsychotics do have varying degrees of adverse effects,^13^ which can limit functionality and quality of life.^14^ Moreover, treatment-resistance rates are about 20% in patients with first-episode and 40% in patients with multi-episode schizophrenia.^15^ Finally, recovery rates remain low, being about 22% in patients with first-episode schizophrenia^16^ and 11% in patients with multi-episode schizophrenia,^17^ and by themselves, antipsychotics cannot give people friends, romantic partners, or jobs, as there are “no skills in pills.” However, through treatment-related symptomatic stability, antipsychotics can enable people living with schizophrenia to take fuller advantage of psychological, psychosocial, and supported education and employment interventions to help them achieve their life goals.^18^

Taken together, this article rebuts held opinions that antipsychotics “don’t work” through an evaluation of meta-analyses, clinical trial data, and real-world effectiveness studies, showing that antipsychotics not only effectively work comparably to—or better than—many standard treatments used in general medicine, but are essential in improving both symptoms and long-term outcomes in people living with schizophrenia.

## Mechanism of action, balancing benefits, and risks

Antipsychotic medications primarily act by modulating dopaminergic transmission, particularly through antagonism or partial agonism of postsynaptic D2 dopamine receptors in the associative striatum pathway, which is thought to underlie the positive symptoms of schizophrenia.^19^^,^^20^ However, the pharmacology of these drugs is far more nuanced. While first-generation antipsychotics (FGAs) predominantly block postsynaptic dopamine receptors, many second-generation antipsychotics (SGAs) also interact with serotonin (5-HT2A), histamine (H1), adrenergic (α1), and cholinergic receptors.^19^ Clozapine, in particular, exhibits a unique receptor profile^21^and remains the gold standard for treatment-resistant schizophrenia.^22^

FGAs can effectively reduce positive symptoms but are often associated with neuromotor side effects. SGAs have broader receptor activity with improved tolerability, but some have relevant cardiometabolic risks, including weight gain and glucose and lipid abnormalities.^23^ Long-acting injectable antipsychotics (LAIs) have emerged as a valuable strategy to address the challenge of nonadherence, a major contributor to relapse, by providing sustained drug release and improving treatment continuity.^24^^,^^25^ Meta-analyses across different study designs, including randomized controlled trials, cohort studies, and pre-post or mirror image studies, have demonstrated an 8–56% reduction in relapse/hospitalization rates with LAIs compared to oral formulations,^26^ with particular superiority in real-world studies and those using patients as their own controls.^26^^,^^27^ Despite these benefits, underutilization of LAIs persists due to clinician hesitancy and perceived patient resistance.^25^^,^^28^

Nevertheless, both acute and long-term adverse effects are a real concern.^13^ FGAs, especially high-potency FGAs, such as haloperidol, are associated with considerable rates of neuromotor side effects, such as acute dystonia, parkinsonism, akathisia, and tardive dyskinesia.^29^^–^^32^ SGAs tend to have more favorable neuromotor profiles but pose cardiometabolic risks, including weight gain, insulin resistance, and dyslipidemia, as well as metabolic syndrome,^33^^,^^34^ each a risk factor for cardiovascular illness, the most common cause of mortality in people with schizophrenia.^35^ Some FGAs and some SGAs are associated with prolactin elevation and related sexual dysfunction^36^ and possibly an increased risk of breast cancer.^37^^,^^38^

Strategies to mitigate these risks include selecting or switching to agents with lower cardiometabolic (eg, aripiprazole, brexpiprazole, cariprazine, lumateperone, lurasidone, ziprasidone) ^39^^,^^40^ or other side effect burden, using the lowest effective dose, but without undercutting it,^41^^,^^42^ monitoring cardiometabolic parameters regularly, using treatments targeting metabolic syndrome parameters,^43^^–^^45^ and incorporating lifestyle interventions.^39^ LAIs can also help maintain therapeutic levels while reducing peak trough fluctuations that contribute to side effects.^46^

## Efficacy and effect sizes: How well do antipsychotics work?

Importantly, however, the benefits of antipsychotic treatment overall outweigh the risks considerably when viewed generally from an individual and population health perspective.^4^ Moreover, premature discontinuation of treatment—often driven by challenges with illness insight and/or insufficiently managed adverse effect burden—is a major driver of relapse, hospitalization, and suicide risk.^47^

Benefits and disadvantages of treatments in medicine are most meaningfully quantified using statistical measures called effect sizes. These metrics provide a standardized way to compare the magnitude of a treatment’s efficacy or effectiveness or adverse effect risk across different medications, studies, and even conditions.^48^ The most widely used effect size in psychiatry for continuous outcomes, such as change in symptoms or blood test measures, is the standardized mean difference (SMD).^49^ SMD is typically reported as Cohen’s d effect size, whereby the number above or below zero (ie, no difference) indicates either the magnitude of increase or decrease in the value compared to a control condition or to baseline when comparing outcomes within a treatment group. By convention, an effect size of 0.2 is considered small, 0.5 is considered medium, and 0.8 and above is considered large.^49^ The advantage of SMDs over weighted mean differences (WMDs), which can only be calculated for studies using the exact same rating scale, is that effects can be pooled across different rating instruments, as the difference between the groups is expressed as the amount of standard deviation units, which can be added and for which mean values can be calculated.

For the continuous outcome time to event (eg, time until relapse), hazard ratios (HRs) are typically used, whereby the decimal value above or below 1 (ie, no difference) indicates the percent risk that is increased or decreased compared to a control condition. For categorical outcomes, such as response, remission, recovery, or relapse, the absolute experimental event rate (EER) is compared with the control event rate (CER), resulting in relative effect size measures, such as odds ratios (ORs) or risk ratios (RRs).^48^ The advantage of RRs is that they can be converted to a risk difference (RD) and then to either the number needed to treat (NNT) for benefits or the number needed to harm (NNH) for negative effects, whereby the resultant full number represents the number of people one needs to expose to the experimental agents to get one additional good (NNT) or one additional bad outcome compared to the control condition.

Examining the question of whether or not antipsychotics work, these metrics provide a powerful and interpretable comparative evidence base, which can demonstrate that antipsychotics not only work but are even among the most effective pharmacological treatments in medicine. Clearly, not everyone improves and antipsychotics are also associated with adverse effects that can be bothersome or even dangerous, but this is also a reality for common medications used in somatic medicine.

In a review of meta-analysis results of antipsychotic efficacy, Leucht et al. compared the results using 13 different effect size measures.^48^ Among those most commonly used, the authors also examined mean difference (MD); SMD; NNT derived from SMD; OR, RR, and RD derived from SMD; drug response; and placebo response in percent. Applying these indices to meta-analyses comparing antipsychotic drugs with placebo for acute schizophrenia, the authors reported a difference of all antipsychotics pooled versus placebo (105 trials, *n* = 22,741 participants) of MD 9.4 (95% confidence interval (CI): 8.4, 10.2) Positive and Negative Syndrome Scale (PANSS) total points, SMD 0.47 (95%CI: 0.42, 0.51), NNT 5 (95%CI: 5, 6), OR 2.34 (95%CI: 2.14, 2.52), RR 1.67 (95%CI: 1.59, 1.73), RD 20% (95%CI: 18, 22), and proportion of patients improved on drug 50% (48, 52) compared to the proportion of patients improved on placebo (30%). Taken together these indices, plus others less often used in publications, indicated a substantial, but not a large superiority of antipsychotics versus placebo. The authors noted that the chronicity of the patients in most of the trials should be considered, as people with first-episode and early-phase illness may experience better antipsychotic response. ^50^^,^^51^

**Figure 1.**
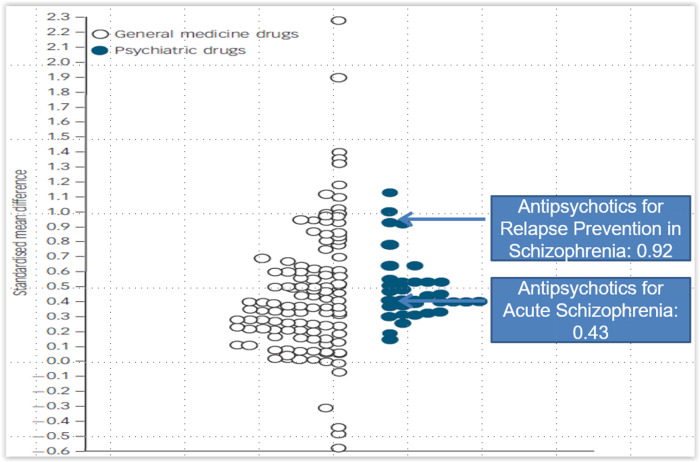
Standardized effect sizes for efficacy of psychiatric and medical drug treatments versus placebo. Adapted from Leucht et al.^52^.

These effect sizes become more powerful when contextualized across medical disciplines and their drug classes. In a review of results from 94 meta-analyses, including 48 drugs in 20 medical diseases and 16 drugs in 8 psychiatric disorders, Leucht et al.^52^ observed that while some general medical drugs had clearly higher effect sizes than psychotropic medications, psychiatric drugs were not generally less efficacious than medical drugs. This finding was subsequently confirmed.^53^ Looking specifically at antipsychotics for people with schizophrenia, the effect size for the improvement of total psychopathology in patients with an acute exacerbation of schizophrenia was SMD = 0.43 versus placebo, which was similar to the median effect size across all medical drugs examined.^52^ However, while acute symptom reduction is critical, the long-term effectiveness of antipsychotics is even more compelling. One of the most replicated findings in psychiatric research is that sustained antipsychotic treatment substantially reduces the risk of relapse in patients with schizophrenia. Thus, when patients responding to antipsychotics were continued on the same antipsychotic or discontinued, the effect size more than doubled from an SMD of 0.43 to an RR-derived SMD of 0.92 for relapse prevention^52^ (Figure 1). This difference in relapse rates, which was not related to the speed of antipsychotic discontinuation,^54^ translated into an NNT of 3, with NNTs <10 being generally clinically relevant and ≤ 5 being strongly clinically relevant.^55^

When comparing the meta-analysis efficacy of medical drugs in the prevention of a negative medical outcome with an NNT of 3 for the prevention of relapse with antipsychotics, it becomes evident that the effect size for antipsychotics in schizophrenia is 10–20-fold stronger (with smaller NNTs indicating greater efficacy)^52^ (Table 1). Specifically, the NNT for the prevention of a major cardiovascular disease event with statins and angiotensin-converting enzyme (ACE) inhibitors was 25 and 67 for aspirin. Similarly, the preventive effect for mortality in type 2 diabetes with metformin was NNT = 31. In addition to antipsychotics having a stronger preventive effect relative to placebo, in absolute numbers, more patients with the disease have the bad outcome prevented with antipsychotics. This advantage in absolute numbers is due to the fact that negative cardiovascular and mortality events were relatively less frequent (6–18% on placebo vs 2.7–14% on drug) than schizophrenia relapses (57% on placebo vs 22% on antipsychotics) (Table 1).

**Table 1.** Efficacy of Antipsychotics and Common Medical Medications in Preventing a Negative Outcome

Treatment	Studies	Patients	Outcome	PBO	Drug	ARD	**NNT** ^a^	**SMD** ^b^
Antipsychotics^c^	62	6392	Schizophrenia relapse	57.0%	22.0%	38%	**3**	**0.92**
Statins^d^	14	90,056	Major CVD event	18%	14%	4%	**25**	**0.15**
ACE inhibitors^d^	5	18,229	CVD events	18%	14%	4%	**25**	**0.16**
Metformin^d^	11	12,840	Type 2 diabetes mellitus mortality	6.0%	2.7%	2.3%	**31**	**0.03**
Aspirin^d^	16	17,000	Secondary prevention of severe CVD events	8.2%	6.7%	1.5%	**67**	**0.12**

Abbreviations: ACE, angiotensin-converting enzyme; ARD, absolute risk difference; CVD, cardiovascular disease event; NNT, number needed to treat; SMD, standardized mean difference.

a Smaller number indicates greater medication efficacy;

b Larger number indicates greater medication efficacy;

c Based on Leucht et al.^52^;

d Based on Leucht et al.^53^.

## Beyond symptoms, treatment persistence, and relapse prevention

However, the benefits of antipsychotics extend beyond acute symptom improvement, maintenance of the effect, and prevention of relapses or hospitalizations. While acute symptom reduction is a basic need and the long-term effectiveness of antipsychotics is critical, functional outcomes and beneficial effects on longevity are even more compelling.

In a nationwide Swedish database study with a within-subject design, the risk of sickness absence or disability pension during antipsychotic use was compared with antipsychotic nonuse during a maximum of 11 years of follow-up.^56^ Among the cohort of patients with first-episode nonaffective psychosis (*n* = 21,551; age range: 16–45 years), 45.9% had work disability during the median length of follow-up of 4.8 years. Altogether, the risk of work disability was significantly lower during use compared with nonuse of any antipsychotic (adjusted hazard ratio [aHR] = 0.65, 95%CI = 0.59–0.72), with the greatest benefit for LAIs (aHR = 0.46, 95%CI = 0.34–0.62). aHRs were similar during the periods of <2 years, 2–5 years, and > 5 years since diagnosis, indicating that ongoing antipsychotic treatment was associated with about 30–50% lower risk of work disability versus nonuse of antipsychotics in the same individuals, which held true even beyond 5 years after first diagnosis, being highly important for the promotion of functional recovery.

Additional real-world evidence supports improvements in functioning and legal outcomes. A recent study of 3166 adults (mean age 38.6 ± 12.6 years), 76.5% with schizophrenia-spectrum disorders in California’s forensic state hospital system who were deemed incompetent to stand trial, showed that as many as 86.5% of them were successfully restored to competency, with 98.8% discharged on antipsychotic medications.^57^ Patients treated with antipsychotic monotherapy demonstrated higher restoration rates compared to those requiring additional mood stabilizers, indicating that more complex or severely ill patients require additional interventions. These findings underscore the beneficial role that antipsychotics can play beyond reducing psychotic symptoms, aiding the restoration of humanistically relevant functional capacity for participation in legal proceedings. These functional improvements can then extend beyond the courtroom, facilitating reintegration for individuals with severe mental illness into the community.

In a related study, post-discharge outcomes among 4056 adult Florida Medicaid enrollees with schizophrenia (69%) or bipolar disorder (31%) were examined.^58^ While altogether 1263 participants (31%) were arrested at least once during follow-up, those adhering to outpatient treatment had a significantly lower risk of any arrests (misdemeanor or felony) and of misdemeanor arrests. Among patients with schizophrenia-spectrum disorders, engagement in outpatient services—including medication adherence—significantly reduced criminal justice involvement.

Finally, the gold-standard outcome in medicine is the reduction of mortality risk. This outcome is particularly relevant for people with schizophrenia who experience an enormous amount of years of potential life lost of as many as 15.37 years (95% CI 14.18–16.55), being third only to commonly comorbid substance use disorders (20.38 years [95% CI 18.65–22.11]) and eating disorders (16.64 years [95% CI 7.45–25.82]).^59^ In this context, recent studies are highly relevant that did not only confirm an increased mortality risk for people with schizophrenia compared to the general population and other mental disorders, but demonstrated that antipsychotic treatment was able to significantly reduce this increased risk.

**Figure 2.**
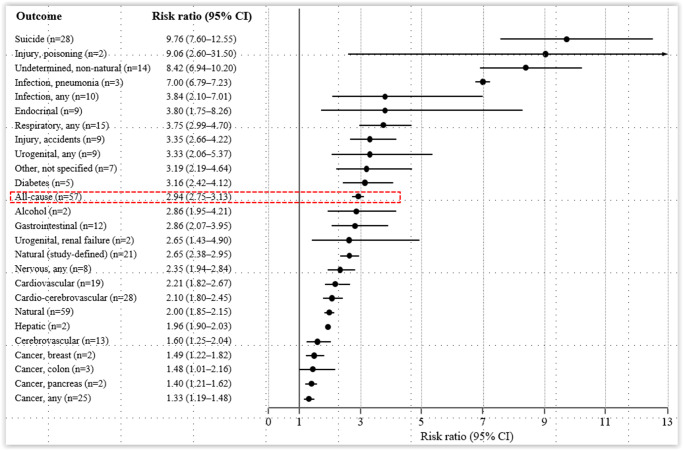
All-cause and specific-cause mortality estimates in people with schizophrenia compared to the general population. CI, confidence interval. Based on Correll et al.^60^.

**Figure 3.**
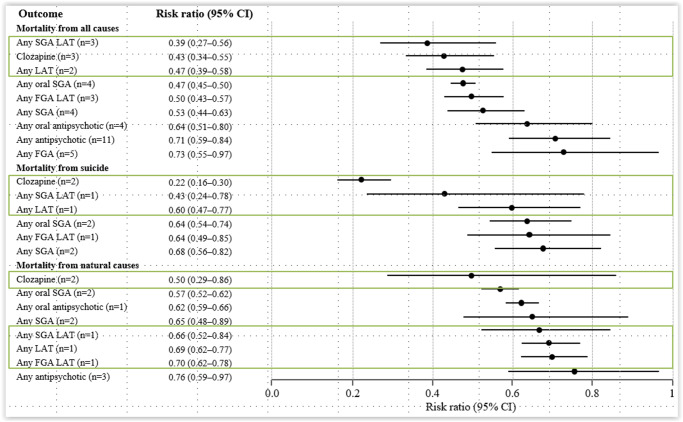
Reduction in all-cause and specific-cause mortality in people with schizophrenia dependent on antipsychotic versus no antipsychotic treatment. CI, confidence interval; FGA, first-generation antipsychotic; LAT, long-acting therapy; SGA, second-generation antipsychotic. Based on Correll et al.^60^.

For example, in a meta-analysis of 135 studies, including 4,536,447 people with schizophrenia, 1,115,600,059 general population controls, and 3,827,955 other psychiatric illness controls, all-cause mortality was significantly increased in people with schizophrenia versus any non-schizophrenia control group (RR = 2.52, 95%CI: 2.38–2.68) (Figure 2). ^60^ The increased mortality risk was most pronounced in people with first-episode (RR = 7.43, 95%CI: 4.02–13.75, *n* = 2) and incident (ie, earlier-phase) schizophrenia (RR = 3.52, 95% CI: 3.09–4.00, *n* = 7) compared to the general population. The specific-cause mortality was highest for suicide/injury/poisoning/undetermined non-natural cause (RR = 9.76–8.42). Compared to individuals with schizophrenia aged ≥40 years old, those aged <40 years old had significantly increased all-cause and suicide-related mortality, and comorbid substance use disorder significantly increased all-cause mortality (RR = 1.62, 95% CI: 1.47–1.80). However, importantly, antipsychotics were protective against all-cause mortality compared to no antipsychotic use (RR = 0.71, 95%CI: 0.59–0.84, *n* = 11), with largest effects for SGA LAIs (RR = 0.39, 95%CI: 0.27–0.56), clozapine (RR = 0.43, 95%CI: 0.34–0.55), any LAI (RR = 0.47, 95%CI: 0.39–0.58), and any SGA (RR = 0.53, 95%CI: 0.44–0.63) (Figure 3). ^60^

Similarly, a Finnish nationwide register-based study sought to determine factors associated with mortality and to demonstrate their impact on expected life-years in patients with schizophrenia.^61^ In these analyses, factors that significantly increased all-cause mortality were cardiovascular disease (HR: 2.41, 95%CI: 2.34, 2.49), liver disease (HR: 1.98, 95%CI: 1.78, 2.21), renal disease (HR: 1.63, 95%CI:1.56, 1.70), longer duration of previous hospitalizations (HR: 1.96, 95%CI: 1.90, 2.02), diabetes (HR: 1.40, 95%CI:1.35, 1.45), history of switching antipsychotics, likely indicating instability (HR: 1.39, 95%CI: 1.35, 1.44), history of substance abuse (HR: 1.38, 95%CI: 1.30, 1.46), and ever use of benzodiazepines (HR: 1.12, 95%CI: 1.09, 1.16). Conversely and importantly, factors that significantly reduced all-cause mortality were use of antipsychotics (HR: 0.46, 95%CI: 0.45, 0.47), ever use of lipid-modifying agents (HR: 0.71, 95%CI: 0.68, 0.73), antidepressant use (HR: 0.87, 95%CI: 0.85, 0.90), and lithium use (HR: 0.90, 95%CI: 0.86, 0.95).^61^

These expected and general-population-consistent findings of cardiovascular disease and diabetes being associated with an increased mortality risk in people with schizophrenia, combined with a significant protective effect of antipsychotics, have led to something called “mortality paradox.”^62^ This seeming paradox is created by the fact that antipsychotics can have adverse cardiovascular and diabetes effects, especially clozapine, which was one of the most used mortality-reducing agents. This seeming paradox has been resolved, however, by a Finnish database study using a within-subject design.^63^ In this study, adults aged <65 years diagnosed with schizophrenia were subdivided into 4 cohorts based on cardiometabolic drug use during the follow-up period, namely statin (*n* = 14,047), antidiabetic (*n* = 13,070), antihypertensive (*n* = 17,227), and beta-blocker (*n* = 21,464) users. Results suggested an indirect, mediating protective effect of antipsychotics use via not only reduced illness severity, psychosis-related stress, and potentially improved healthy lifestyle behaviors, but antipsychotic use also increased persistence of secondary preventive medical drugs, offsetting the potential increased cardiovascular risk of some antipsychotics, including clozapine and LAIs, which were each most protective against premature mortality^60^^,^^61^ and were also associated with greater adherence to cardiometabolic drugs. Specifically, when ranking antipsychotics regarding reducing discontinuation of all cardiometabolic drug categories the most protective was clozapine (aHR range = 0.34–0.55), followed by olanzapine (aHR = 0.43–0.71).^63^

Taken together, the summarized consistency across metrics and outcomes confirms that antipsychotics provide a measurable enabling benefit, not merely conferring sedation or behavior control as sometimes claimed by critics. The efficacy of antipsychotics includes acute reduction in symptoms and prevention of relapse, but further extends across symptom domains, reaching also functional areas, reflecting their true efficacy and effectiveness, thereby not just representing nonspecific trial participation or placebo effects and not being due to sedative properties or even “chemical restraint” effects, as has sometimes been claimed.

## Integrating antipsychotic use with psychosocial care and global guidelines

While antipsychotic medications are foundational in the treatment of schizophrenia, they are most effective when embedded within a broader biopsychosocial framework. Comprehensive care includes not only pharmacological treatment but also individual and family psychoeducation, cognitive-behavioral therapy (CBT), supported employment, social skills training, and case management services.^10^

International and national guidelines consistently advocate for multimodal interventions, emphasizing also person-centered care, early intervention, and integration of pharmacological and psychosocial therapies.^64^^,^^65^ These models combine the backbone of antipsychotic treatment with psychotherapy, supported education/employment, and assertive outreach to optimize functional outcomes. Evidence suggests that early integrated interventions, also called coordinated specialty care, for people with first-episode and early-phase illness improve engagement, reduce relapses, and increase the likelihood of recovery compared to usual community care,^66^ at least when individuals with schizophrenia receive this specialty care.

Moreover, recent clinical care models increasingly emphasize digital health tools, measurement-based care, and shared decision-making as core components of modern schizophrenia management.^67^^–^^69^ Digital adherence tools (eg, smart pill bottles, digital blister packs), symptom tracking apps, and telepsychiatry offer scalable ways to enhance the monitoring and personalization of care. Thus, ultimately, antipsychotics are most impactful when used as part of a comprehensive treatment strategy that respects patient autonomy, addresses environmental and social factors, and targets recovery, not merely symptom suppression.

## Conclusions

While no single treatment is universally effective, the overall evidence unequivocally supports the use of antipsychotics as essential components of care for people with schizophrenia. Based on the reviewed evidence above, the evidence is clear and consistent: antipsychotics work in people with schizophrenia. Their efficacy, demonstrated through dozens of meta-analyses, randomized trials, and real-world cohort studies, and measured by multiple effect size indices and long-term outcomes, is robust and comparable to, or exceeds, that of many accepted treatments in internal medicine. Importantly, antipsychotics do more than reducing symptoms. They restore stability, prevent relapse, enable functional recovery, reduce the likelihood of incarceration, help patients regain competency in legal contexts, and reduce the increased risk of premature mortality due to any cause, suicide, and common medical conditions.

However, these advantages are obviously—like for medical conditions and treatments—based on group mean values and proportions. Hence, not all patients with schizophrenia can and will benefit sufficiently, or not across all desired domains or not at all, from antipsychotic treatment. Moreover, some acute or long-term side effects will limit the use and benefits of antipsychotics for relevant subgroups of patients. This situation means that better treatments for individuals and subgroups of patients with schizophrenia still need to be sought and that individualized care is in dire need. Moreover, best outcomes occur when pharmacological treatment is embedded in a framework of psychosocial support and individualized care.

Based on this evidence, mental health professionals, policymakers, and advocates should feel confident in asserting that antipsychotics work for people with schizophrenia. Their continued use—combined with strategies to enhance adherence, reduce side effects, and promote recovery—is not a matter of debate but of responsibility. Dismissing the value of antipsychotics on the basis of side effects or ideological positions undermines the lived experience of millions who can benefit from them. Such a position also misleads policymakers, families, and even patients, who may forego potentially life-saving treatment due to misinformation. Antipsychotics, especially when paired with psychosocial and psychological interventions, are foundational to current schizophrenia care.

The path forward is not to question whether antipsychotics work, but how to best deploy them safely, equitably, and in alignment with patient goals. A truly recovery-oriented system must integrate pharmacological and psychosocial care, address stigma, and empower individuals to reclaim meaningful lives. We must shift the public discourse from polarized skepticism to informed understanding. The lives of patients—and the integrity of psychiatric practice—depend on it.
